# Preparation of Synbiotic Yogurt Sauce Containing *Spirulina platensis* Microalgae Extract and Its Effect on the Viability of *Lactobacillus acidophilus*

**DOI:** 10.1155/2023/8434865

**Published:** 2023-12-26

**Authors:** Hamidreza Kazemeini, Asghar Azizian, Katayoun Ahmadi

**Affiliations:** ^1^Department of Food Hygiene, Faculty of Veterinary Medicine, Amol University of Special Modern Technologies, Amol, Iran; ^2^Department of Food Hygiene and Quality Control, Faculty of Veterinary Medicine, University of Tehran, Tehran, Iran; ^3^Department of Food and Aquatic Health, Faculty of Veterinary Medicine, Ferdowsi University of Mashhad, Mashhad, Iran

## Abstract

**Background:**

Preparing a healthy and practical substitute for mayonnaise and reducing the complications caused by its consumption are two of the concerns of the producers of this product. Therefore, this study was conducted with the aim of evaluating the possibility of producing synbiotic yogurt sauce prepared with *Spirulina platensis* microalgae extract (SPAE) as a valuable and alternative product for mayonnaise.

**Materials and Methods:**

After preparing yogurt from fresh cow's milk, synbiotic yogurt sauce was prepared according to the formulation, and the effect of SPAE at the rate of 0.5, 1, and 2% on the viability of the probiotic bacteria *Lactobacillus acidophilus* was evaluated, and chemical, rheological, and sensory tests were carried out in the storage period (35 days).

**Results:**

The highest viability rate of *L. acidophilus* was related to the treatment containing 2% of SPAE with 1.31 log CFU/g reduction (from 9.02 log CFU/g on the first day to 7.71 log CFU/g on the final day) and 1% of SPAE with 2.98 log CFU/g reduction, respectively, which were significantly more effective than other treatments (*P* < 0.05), and it was found that the viability rate increases with the increase in the percentage of the prebiotic composition. There was also a significant difference between the treatments in the simulating conditions of the digestive system, and the viability of *L. acidophilus* in the treatment containing the prebiotic composition increased (*P* < 0.05). According to the results, during storage, in the presence of microalgae, acidity increased, and pH, viscosity, and sensory properties decreased compared to the control group. Upon analyzing the results, it was found that the addition of the prebiotic composition of SPAE, which is known as a functional product, led to a partial improvement in its properties. Therefore, the use of this alga, while benefiting from its medicinal and therapeutic properties, increases the viability rate of probiotic.

## 1. Introduction

The role of food in the body, in addition to providing the necessary substances, is to help treat and prevent the spread of chronic diseases such as cancer, cardiovascular diseases, osteoporosis, and other cases, and for this reason, it is very important in maintaining the health of the body. This indicates the necessity of consuming functional foods and its presence in the daily diet of every person [1, 2]. These products may contain soluble fibers, omega-3 fatty acids, unsaturated fatty acids, conjugated linoleic acid, vitamins and minerals, some proteins, peptides and amino acids, phospholipids, and natural antioxidants, but the most important feature of these products is the presence of probiotics and prebiotics [3].

Probiotics exert their health-giving effects by maintaining, improving, and balancing the intestinal microbiota [4]. Consequently, various studies have been conducted so far regarding their use in food products, and the results indicate their health-enhancing effects [5] and increasing the demand and popularity of probiotic products among consumers [6, 7].

Microorganisms used as probiotics should be resistant to bile and stomach acids, can settle in the human digestive system, be nonpathogenic, and have proven effectiveness and safety [8]. In order to create the maximum health-giving effect and the best performance in the consumer, it is necessary to have at least 10^6^ CFU/g or mL of the probiotic species at the time of consumption in the probiotic products. Hence, maintaining the viability and ensuring the viability of probiotic bacteria is a big challenge for producers and food industry activists [8]. Adding prebiotics to probiotic compounds results in the production of synbiotic products, which are in the category of functional foods [9].

One of these prebiotic compounds is *Spirulina platensis*, which is a type of blue-green microalgae, and belongs to the branch of cyanobacteria and the Oscillatoriaceae family [10]. It is a rich source of vitamins, minerals, proteins, amino acids, and essential fatty acids [6]. In the use of *Spirulina platensis* powder as a dragée to enrich snacks and to check the durability of snacks enriched with dragées [11] and the use of spirulina biomass on the technological and nutritional quality of pasta and bread [12], there are studies that have used spirulina so far.

Currently, due to the increase in the consumption of various sauces, which causes a high amount of calories and complications such as obesity and cardiovascular diseases, producers are facing challenges to find alternatives with reduced calories [13]. Lately, a flavored sauce based on yogurt is prepared under the name of yogurt sauce, which is very popular and in demand among consumers, and in addition to reducing the number of calories (compared to salad dressings and mayonnaise and the complications caused by its consumption), it has benefits. It also contains yogurt, and by adding other additives, positive changes can be made in its sensory properties, and probiotic sauces with health-giving properties can also be produced [13, 14]. According to what was said, the purpose of this research was to produce synbiotic yogurt sauce containing *Spirulina platensis* and to investigate its effect on the viability of *L. acidophilus* bacteria under refrigerated and digestive simulating conditions and to investigate sensory, rheological, and chemical properties.

## 2. Materials and Methods

### 2.1. Preparation of SPAE

Initially, 150 g of dried seaweed with the Green Sea brand was purchased from a reliable supply center and crushed by a grinder (Moulinex, Spain). Then, it was mixed with water and placed on a shaker (T&N, China) for 48 h. Afterward, it was filtered, and this step was repeated by adding 1 L of water. Toward concentrate and dry, the extract was placed in a Fore device at 40 ± 1°C for one week. The dried extract was kept at a temperature of 4°C in light-proof glasses [15].

### 2.2. Probiotic Preparation

The culture of lyophilized *L. acidophilus* (LA-5) prepared by Christian Hansen (Horsholm, Denmark) was placed in sterile MRS Broth (Merck, Germany) culture medium at 37°C for 24 h in an anaerobic condition incubated. Recultivation was carried out for 48 h to obtain the maximum number of active probiotics. Then, the cell mass was collected by a refrigerated centrifuge (Eppendorf AG, Germany) at 4000 rpm for 15 minutes, and in duplicate with sterile physiological serum, 0.9% was washed, and the bacterial suspension was calculated with a concentration of 1 × 10^9^ CFU/mL [7].

### 2.3. Preparation of Probiotic Yogurt Sauce and Treatments

**Figure 1:**
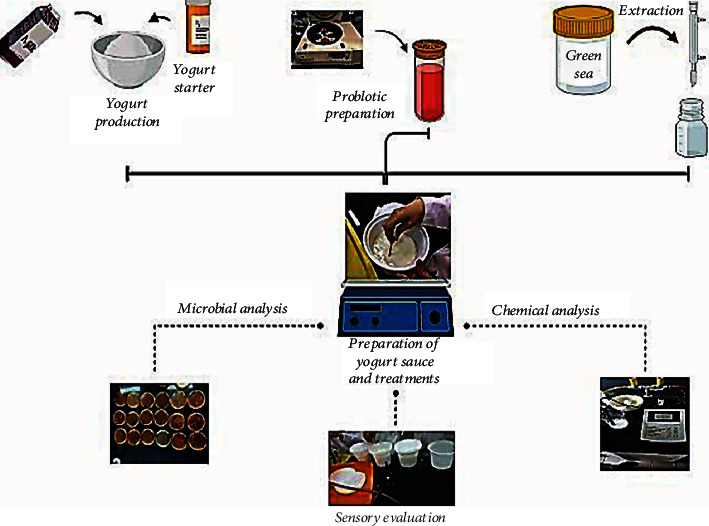
Preparation of probiotic yogurt sauce and treatments.

Figure 1 shows a summary of the activities carried out. First pasteurized and homogenized low-fat milk was prepared, then boiled for 5 minutes at a temperature of 100°C, and at a temperature of 40-45°C, the starter treatment (mixed culture of *Streptococcus thermophilus* and *Lactobacillus delbrueckii* subsp. *bulgaricus*) by Christian Hansen (Horsholm, Denmark) was performed. It was placed in an incubator under suitable conditions, and after clot formation and reaching pH = 4.4, cooling was done. Then, it was filled and sealed in suitable sterile polyethylene containers and kept at refrigerator temperature [6].

To prepare samples of yogurt sauce according to the formulation in Table 1, first sugar, salt, mustard, vinegar, and citric acid are mixed with water, and then, the mentioned amount of prepared yogurt is added to it, and with the help of a mixer (3M43, KENWOOD, England) it was well mixed. While stirring, the carrageenan gum powder was gradually added, and then, the egg was added to the mixture and completely homogenized to obtain a uniform composition. In the last step, corn oil was added drop by drop and continuously. Yogurt sauce samples prepared in the laboratory in Ben Marie (Memmert, Germany) were pasteurized at 65°C for 30 minutes. Then, the treatments were prepared according to Table 2, and the samples were kept at 4°C for 35 days. Physicochemical, microbial, rheological, and sensory tests were performed in 3 repetitions on days (0, 7, 14, 21, 28, and 35) of storage [6, 16].

### 2.4. Viability of *L. acidophilus* in Yogurt Samples

*L. acidophilus* was counted on the study days. After preparing the series of dilutions, it was cultured in MRS bile agar medium (Merk, Germany) in three replicates and kept in an incubator at a temperature of 37°C for 72 h. The counting results were reported in terms of CFU/g [3].

### 2.5. Viability of *L. acidophilus* in Conditions Simulating the Digestive Tract

Artificial gastric solution of 1.12 g of potassium chloride (Merk, Germany), 2 g of sodium chloride (Merk, Germany), 0.11 g of calcium chloride (Merk, Germany), and 0.4 g of basic potassium phosphate (Merk, Germany) in a volume of one liter was prepared. The amount of 1 g of sample was added to 50 mL of gastric solution, and it was kept at a temperature of 37°C in a shaker incubator (GEL, model 3031, Germany) at certain times (0, 30, 60, 90, and 120 minutes) and cultured. The pour plate was examined in MRS agar medium (Merk, Germany). In the simulation of intestinal conditions, 1.95 g of pancreatin (Merk, Germany) and the same amount of bile salt (Merk, Germany) were added to the containers containing the prepared stomach solution, and its pH was close to 1 normal (Merk, Germany). It was set to neutral and kept at 37°C in a shaker incubator (GEL, model 3031, Germany) for specific times (0, 60, 120, 180, and 240 minutes). To check the viability rate of bacteria, pour plate culture was performed on MRS agar medium (Merk, Germany) [17].

### 2.6. pH Measurement

The pH of the samples was measured using a pH meter (Metrohm, Switzerland). The amount of 10 g of the homogenized samples was poured into the beaker and measured by a pH meter which was initially calibrated at 20°C by buffers 4 and 9 [18].

### 2.7. Acidity Measurement

The amount of titratable acidity of the samples was measured with the help of the titration method. The amount of 10 g of the sample was dissolved with distilled water at a temperature of 45°C and brought to a volume of 100 mL. Then, 10 mL of it was titrated with sodium hydroxide in the presence of phenolphthalein reagent until a stable pink color appeared, and the acidity was determined in terms of lactic acid percentage [18].

### 2.8. Viscosity Measurement

Using a Brookfield viscometer, the apparent viscosity of the samples was measured on the first day and the last day of the study (day 35) using a Brookfield rotary viscometer made in the United States at a temperature of 25°C. The viscosity of different samples was compared with each other at a cutting speed of 50/s. The flow behavior of yogurt sauces produced at different cutting speeds was investigated [19].

### 2.9. Sensory Evaluation

The samples were prepared according to the treatment table (Table 2) and randomly coded. Sensory characteristics (taste, texture, and color) were evaluated by 10 trained evaluators using a 5-point hedonic rating scale, average, bad, and very bad [20].

### 2.10. Color Measurement

The color components were measured using a Hunt lab (Hunter Lab Color Flex) device. The colorimeter was first calibrated using a black and white screen, and then, the samples were transferred into the machine and tested. Based on light reflection, color indices including *L*^∗^ (brightness or full light), *a*^∗^ (green), *b*^∗^ (yellowish-blue), and Δ*E*^∗^, which indicates the total color changes, were measured [6].

### 2.11. Statistical Analysis

The research is in the form of a completely randomized design consisting of 4 factors, the type of prebiotic composition (in one level), the percentage of prebiotic addition (in four levels: 0, 0.5%, 1%, and 2%), diversity of probiotic bacteria in one level (*L. acidophilus*), and storage time at six levels (0, 7, 14, 21, 28, and 35 days) accomplished in three replications. One-way analysis of variance was used to analyze the effect of time on the viability of probiotic bacteria in the simulated conditions of the digestive tract. To compare the means for the analysis of the effect of formulation factors and storage period and their mutual effect, a two-way analysis of variance and Duncan's multiple range tests were used at the 95% confidence level. SPSS software version 25 was used. Draw graphs were carried out using office software.

## 3. Results and Discussion

Considering that the viability of probiotic bacteria in food products customarily decreases during storage, the issue of increasing the viability of probiotic bacteria until the time of consumption and in the conditions of the digestive system is of interest to experts and a challenge for producers [21]. Based on this, the current study was conducted to evaluate the prebiotic effect of SPAE on the viability rate of *L. acidophilus* in yogurt sauce samples during 35 days of storage at refrigerator temperature and also investigate its physicochemical, rheological, and sensory properties.

### 3.1. Viability of *L. acidophilus* in Synbiotic Yogurt Sauce during 35 Days of Storage at 4°C

**Figure 2:**
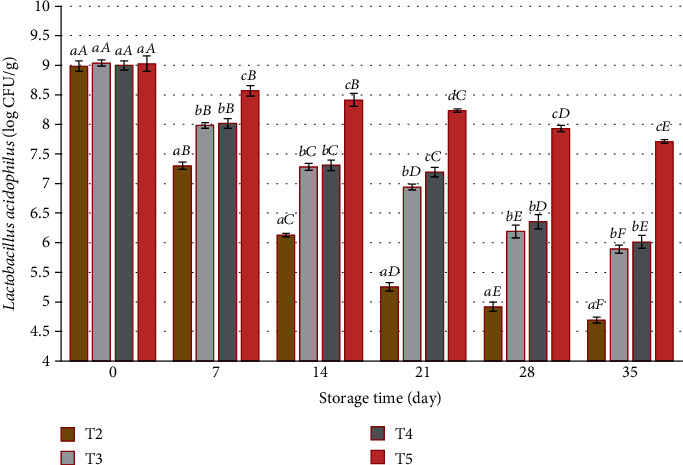
Viability of *Lactobacillus acidophilus* bacteria in synbiotic yogurt sauce during storage. T2: samples containing *L. acidophilus*, T3: samples containing *L. acidophilus* and 0.5% SPAE, T4: samples containing *L. acidophilus* and 1% SPAE, and T5: samples containing *L. acidophilus* and 2% SPAE. Different lowercase letters indicate significant differences between groups (*P* < 0.05), and different uppercase letters indicate significant differences (*P* < 0.05) within days for each treatment.

The viability of *L. acidophilus* in synbiotic yogurt sauce under storage conditions (35 days at 4°C) is shown in Figure 2. The number of live bacteria during the storage period showed a significant decrease in all treatments compared to the first day (*P* < 0.05). Treatments containing SPAE significantly increased the viability of *L. acidophilus* probiotic bacteria (*P* < 0.05), which these results are consistent with the results of a study by Golmakani et al. In this study, the effect of SPAE on the growth of *Lactobacillus casei* in a type of feta cheese was investigated for two months, and the results showed that SPAE is effective in maintaining the viability of probiotic bacteria [22].

In a study by Beheshtipour et al., the effect of adding 2 species of microalgae *Arthrospira platensis* and *Chlorella vulgaris* on the viability of *L. acidophilus* and bifidobacteria in yogurt during a 28-day storage period at refrigerator temperature was investigated. The treatments included 7 groups of yogurt containing three concentrations for each microalga (0.25, 0.50, and 1%) and control. Based on the findings, the addition of microalgae significantly increased the viability of probiotic bacteria during the study. In treatments containing 0.5 or 1% of microalgae, viability was almost higher than 10^7^ CFU/mL until the end of storage in the refrigerator, which is consistent with the results of this study. In this study, it was also found that the increase in the percentage of microalga extract leads to an improvement in viability and that the highest viability rate of probiotic bacteria per day was related to the treatment containing 2% of SPAE (T5) [23].

As expected, at the end of the storage period, the highest viability was related to the treatment containing *L*.*acidophilus* + 2% SPAE (T5) (7.71 log CFU/g), and the lowest viability rate was related to the treatment containing *L. acidophilus* alone (T2) (log CFU/g 4.69) that was compared with a study by Çelekli et al. which is aimed at investigating the effect of SPAE in concentrations (0, 0.25, 0.5, and 1 percent). It is consistent with increasing the growth and activity of probiotic bacteria (*Streptococcus thermophilus*, *Lactobacillus delbrueckii* subsp. *bulgaricus*, *L. acidophilus*, and *Bifidobacterium lactis*) in buttermilk during 21 days [24]. Guldas and Irkin evaluated powdered *Spirulina platensis* on plain yogurt and yogurt containing 10^9^ CFU/g of *Lactobacillus acidophilus*. They reported that the positive effect of *S. platensis* powder on the survival of lactic acid during the storage of yogurt is observed. In all the samples added with spirulina powder, the number of lactic acid was higher than 10^6^ CFU/g, while the control yogurt samples contained fewer amounts of lactic acids at the end of the storage period [25].

The study's findings indicate that it has been determined that the addition of SPAE significantly increased the growth of probiotics compared to the control sample after fermentation and in storage, and in general, SPAE has a great potential to increase the growth of probiotic bacteria and the nutritional content of buttermilk. It is effective [24]. Likewise, in a study by Varga et al., the effect of SPAE on the microflora of *acidophilus bifidus*-*thermophilus* probiotic fermented milk was investigated during storage at two temperatures of 15 and 4°C for 18 and 42 days, respectively. *Acidophilus bifidus*-*thermophilus* milk enriched with spirulina and fermented control was produced using rapid fermentation starter culture as a source of *L. acidophilus*, bifidobacteria, and *Streptococcus thermophilus*, and microbial tests were performed at regular intervals. According to the results, it was found that spirulina had a beneficial effect on the viability of the mentioned bacteria regardless of the storage temperature [26]. While in a study by de Medeiros et al. with the results investigating the effect of SPAE on the growth of *L. acidophilus* during fermentation (37°C, 72 h) in a culture medium, it was found that SPAE improves the growth of *L. acidophilus* and increases metabolic activity, and it is consistent with the results of this study [27]. In a similar study by Fadaei et al., the effect of SPAE on the viability of *L. acidophilus* added to spinach yogurt stored at refrigerator temperature and the starter bacteria *Lactobacillus delbrueckii* subsp. *bulgaricus* and *Streptococcus thermophilus* were investigated in 21 days. It was observed that SPAE was significantly effective on the growth of lactic acid bacteria in probiotic spinach yogurt (*P* < 0.01) and provided a suitable environment for the growth of this group of bacteria, which is consistent with the results of this study [28]. Plus, a study was conducted by Agustini et al. to investigate the effect of adding 1% of SPAE in yogurt on the total number of lactic acid bacteria in it, and according to the results, the yogurt sample containing 1% of SPAE in the total number of acid lactic bacteria had a significant difference with the control sample, which is consistent with the results of this study [15].

The prebiotic effect of compounds such as microalgae in improving the growth of probiotics in food products is related to the compounds present in them. In this regard, a study was conducted by Nowak et al. Commercial prebiotics such as inulin and fructooligosaccharides can significantly increase the growth of probiotics [29]. A study by Ehsani et al. is aimed at producing synbiotic yogurt containing different levels of probiotic bacteria (*L. acidophilus* and *Bifidobacterium lactis*) and commercial inulin extracted from artichoke root (*Cynara scolymus L.*), and it was determined that inulin extracted from artichoke increased the viability of probiotic bacteria in yogurt, and this property was improved by increasing its percentage in yogurt [30]. While in a study by de Medeiros et al., it was also found that the SPAE has a higher prebiotic index than fructooligosaccharides, according to the study by Nowak et al. [27, 29]. In several reports, it has been stated that the viability of probiotic bacteria in yogurt and the nature of compounds made from yogurt are low [31]. But according to the findings of the present study, synbiotic yogurt sauce containing SPAE is a suitable carrier for probiotics because in each sample during the study, there was a suitable amount of probiotics for health-giving effects.

### 3.2. Viability of *L. acidophilus* in Simulated Conditions of the Gastrointestinal Tract

**Figure 3:**
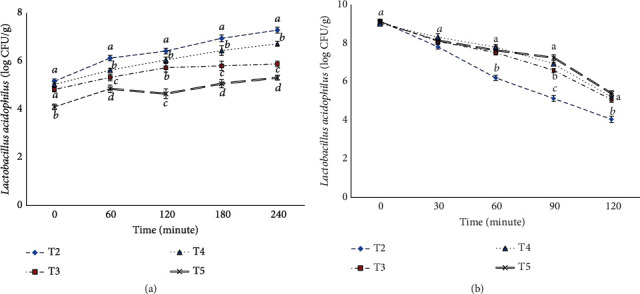
Viability of *Lactobacillus acidophilus* bacteria in simulated intestinal conditions (a) and stomach conditions (b). T2: samples containing *L. acidophilus*, T3: samples containing *L. acidophilus* and 0.5% SPAE, T4: samples containing *L. acidophilus* and 1% SPAE, and T5: samples containing *L. acidophilus* and 2% SPAE. Different lowercase letters indicate significant differences between groups (*P* < 0.05).

What is important when producing food products containing probiotic cells is the ability to survive and maintain the viability of these bacteria, in addition to the length of the production process and storage conditions, but also during transmission through the digestive system. In the conditions of the digestive system, bacteria must resist exposure to stomach acidity and bile salts in the small intestine [32]. For this purpose, the viability of *L. acidophilus* probiotic bacteria in the simulated conditions of the stomach and intestine was evaluated, and its results are shown in Figure 3. Due to the acidic conditions of the stomach during 2 h in all treatments, the number of bacteria showed a significant decrease (*P* < 0.05), which is consistent with a study conducted by Vinderola and Reinheimer. This study is aimed at investigating the composition of 3 probiotics, Bifidobacterium, *L. acidophilus*, and *Lactobacillus casei*, in a type of Argentinian cheese and observed that these mentioned bacteria were compatible with the studied cheese and could survive for up to 3 h in the digestive tract test in simulating gastric juice, and as the environment became more acidic, this amount decreased [33]. As expected, treatments containing SPAE (T3, T4, and T5) were resistant to gastric acid conditions and showed less reduction. In Figure 3, during 3 h of all treatments, the growth of bacteria was somewhat increasing, which results are consistent with a study by Gagliarini et al. [34]. This issue can be due to the prebiotic properties of SPAE and the recovery of live probiotic bacteria when leaving the acidic conditions of the stomach and entering the neutral environment of the intestine. de Medeiros et al. investigated the effect of SPAE along with *L. acidophilus* on the composition of the intestinal microbiota of healthy and middle-aged people through the simulation of the digestive system. Based on the results, SPAE with *L. acidophilus* caused the modification of intestinal microbial flora, which is consistent with the results of this study [27].

In a study conducted by Liu et al., it was found that *Arthrospira platensis* is effective in preventing the growth of harmful bacteria in the intestine of shrimp, modulating the intestinal microbiota, and improving its health and growth performance [35]. After analyzing the results, it was found that the prebiotic SPAE has a good protective effect on the probiotic *L. acidophilus* in the investigated treatments under the conditions of simulating the digestive system, and it increases its viability rate, and this protection increases with increasing the concentration of SPAE in the yogurt sauce.

### 3.3. pH and Acidity

In Table 3, the changes in pH and, in Table 4, the changes in the acidity of synbiotic yogurt sauce containing the probiotic are shown in storage conditions (35 days at 4°C). As can be seen, the trend of pH and acidity in all treatments was decreasing and increasing, respectively. There was no significant difference between treatments until day 21 in terms of acidity. Upon completion of the study, the samples containing SPAE had a lower pH than the samples containing probiotic bacteria alone; this could be due to the nitrogenous substances in SPAE and stimulation of the treatments containing SPAE in acid production [36]. As we expected from the results of this study, the treatment containing 2% SPAE and probiotic bacteria had the lowest pH (3.80), the highest acidity (0.98), and a significant difference with other treatments containing lower concentrations of SPAE. According to the study of Beheshtipour et al., it is due to the buffering capacity in samples containing microalgae. In this study, the effect of adding two species of microalgae, *Arthrospira platensis* and *Chlorella vulgaris*, on the biochemical properties (pH and titratable acidity) of yogurt during a 28-day storage period at refrigerator temperature was investigated. The treatments included 7 groups of yogurt containing control and three concentrations for each microalga (0.25, 0.50, and 1%). According to the results, it was found that pH decreased and acidity increased during the study, which is consistent with the findings of this study [23]. Also, in a study by Çelekli et al., the effect of SPAE in concentrations (0, 0.25, 0.5, and 1%) on the chemical properties (pH and titratable acidity) of buttermilk has been investigated. Based on the results, it was found that the samples containing SPAE had higher titratable acidity levels than the control during the study period, which is consistent with the results of this study [24]. While in a study by Varga et al., the effect of SPAE on the chemical properties of *acidophilus bifidus-thermophilus* probiotic fermented milk during storage at two temperatures of 15 and 4°C for 18 and 42 days, respectively, has been investigated. According to the results, it was found that the pH decreased at a temperature of 15°C but remained constant at a temperature of 4°C during storage [26].

In a study by de Medeiros et al., the effect of SPAE on some chemical properties of the culture medium was investigated. According to the findings, in the presence of SPAE, lower pH values and higher concentrations of acetic, lactic, and propionic acids were observed [27]. Likewise, in a study conducted by Golmakani et al., the effect of SPAE on the titratable acidity of a type of feta cheese was investigated for 60 days. The results showed a higher level of acidity in the samples containing SPAE compared to the control samples [22]. In the comparison of the treatment containing probiotic bacteria and the control, it was also found that at the end of the study, the treatment containing probiotics showed lower pH and higher acidity, which according to the study of Talwalkar et al., the reason is lactose fermentation and acetic acid production by probiotic bacteria [36].

### 3.4. Viscosity

The results of changes in the viscosity of synbiotic yogurt sauce containing *L. acidophilus* kept at refrigerator temperature on days 0 and 35 (the end of the study) are shown in Table 5. As can be seen, in the treatment containing probiotic bacteria alone, due to bacterial activity and increased acid production, increased acidity, and decreased pH, the viscosity decreased, while in the samples containing prebiotic compound (SPAE), due to increased growth and preservation, more viability of probiotic bacteria, more acid production, increase in acidity, and decrease in pH, viscosity has a greater drop, which is consistent with studies by Çelekli et al. [24]. In this study, the effect of SPAE in concentrations (0, 0.25, 0.5, and 1%) on the viscosity of buttermilk was investigated, and according to the findings, it was found that the viscosity values of samples with SPAE decreased during the storage time. Plus, in a finding by Agustini et al., which was conducted, to investigate the effect of 1% SPAE on yogurt, it was found that the yogurt sample containing 1% SPAE had a significant difference in viscosity from the control sample [15].

### 3.5. Color Value

Table 6 shows the color indices for the samples. The value of *a*^∗^ index was in the range of 3.18 to 21.31. The addition of SPAE, which is green, increased *a*^∗^ value (*P* < 0.05). The positive yellowness-blue index (*b*^∗^) indicates the predominance of yellowness in yogurt sauce samples. The value of *L*^∗^ in all investigated treatments was between 50.84 and 51.09, and no significant difference was noted in the treatments (*P* > 0.05). The value of Δ*E*^∗^ indicates the general difference of the measured color parameters between the samples and can be used as a main index to investigate color changes. The Δ*E*^∗^ value changes ranged from 1.05 in the control samples to 4.71 in the samples with 2% SPAE. According to the changes in the overall color index (Δ*E*^∗^), the color difference between the samples containing 2% SPAE and the control sample was significant (*P* < 0.05).

One of the most important visual features in dairy products is color. The color of these products is influenced by various factors, including the type of product and additives [6].

Olson and Aryana also stated that the *b*^∗^ value of our sample is influenced by the level of inoculation; *L. acidophilus* was placed, and the higher inoculation level of *L. acidophilus* made the yogurt darker and yellower [37]. In a research, the possibility of enriching snacks with *Spirulina platensis* powder (SP) as a dragée was investigated, and the results showed that the addition of SP increased flavonoids, total anthocyanin content, vitamins, protein, minerals, essential and nonessential amino acids, and fatty acids including *ω*3 and *ω*6 and had a significant effect on all the color parameters considered by the panels [11]. Khan et al. reported that the color characteristics of mayonnaise formulas enriched with encapsulated vitamin D were not significantly different in *L*^∗^, *a*^∗^, and *b*^∗^ indexes. They explained that the color of the mayonnaise changed from gray to a brighter white, possibly due to increased light-scattering properties. Likewise, the change in the value of *a*^∗^ was attributed to the change in the amount of moisture in different mayonnaise formulas [38].

### 3.6. Sensory Evaluation

**Figure 4:**
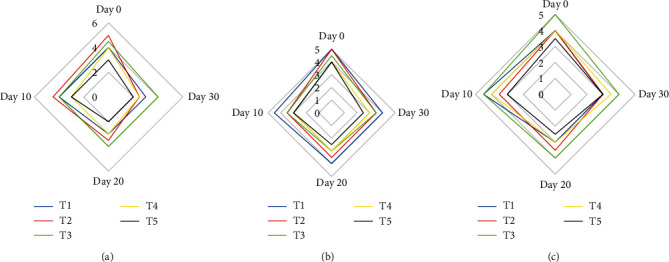
Sensory properties including taste (a), color (b), and texture (c) of different treatments stored in cold storage (4 ± 1°C) for 30 days. T1: yogurt sauce samples, T2: samples containing *L. acidophilus*, T3: samples containing *L. acidophilus* and 0.5% SPAE, T4: samples containing *L. acidophilus* and 1% SPAE, and T5: samples containing *L. acidophilus* and 2% SPAE.

The results of evaluating the effect of SPAE on sensory indicators (taste, color, and texture) of probiotic yogurt sauce kept at refrigerator temperature on days 0, 10, 20, and 30 are shown in Figure 4. As can be seen in the examination of these properties, the trend was decreasing during the study in all treatments, and during the study on all days, the treatment containing probiotic and 2% SPAE had the lowest score. In the taste test, the treatment containing 0.5% SPAE had the highest score. In the examination of color and texture, the number of points decreased with the increase in the percentage of SPAE. In a study by Golmakani et al., the effect of SPAE on the sensory characteristics of a type of feta cheese was investigated over a period of 60 days. The results showed a softer texture in the samples containing SPAE compared to the control samples. However, the samples containing 0.5 or 1% SPAE did not differ significantly from the control samples, which is consistent with the results of this study [22]. In the report of Turk et al. who investigated the possibility of enriching snacks with Spirulina platensis (SP) powder as a dragée, it is stated that the addition of SP resulted in a very good taste score by the panel members [11]. In a study by Grattepanche et al., it was found that increasing the viability of probiotic bacteria in cheese samples due to fermentation and production of acids such as acetic acid had a positive effect on its sensory properties, and the results of this study are consistent with the present study [39]. In a study conducted by Shaden and Abdulsalam, the effect of adding SPAE on the sensory properties of ketchup has been investigated. The results showed the favorable organoleptic properties of the samples and their acceptability for consumers, and the best concentration was reported to be the treatment containing 1% SPAE [40].

## 4. Conclusion

On the whole, the findings of the present study showed that the enrichment of yogurt sauce with *Spirulina platensis* microalgae was significantly effective in improving the viability and survival of probiotic bacteria, which was also observed in the conditions of the digestive tract simulator. Upon analyzing the results, it was found that the application of microalgae had no negative effect on chemical, sensory, and rheological properties. The results of this study can be effective in solving the challenges and concerns of producers, experts, and consumers of beneficial food products in order to increase the viability of probiotics and also use the prebiotic properties of spirulina microalgae.

## Figures and Tables

**Table 1:** Yogurt sauce formulation.

Raw material	Yogurt	Oil	Egg	Sugar	Water	Salt	Mustard	Citric acid	Carrageenan gum
Amount (%)	35	30	9	5	18.5	1.2	0.3	0.03142	1

**Table 2:** List of treatments in the present study.

Treatment (T)	Prebiotic compound	Probiotic bacteria
T1	—	—
T2	—	*L. acidophilus*
T3	0.5% SPAE	*L. acidophilus*
T4	1% SPAE	*L. acidophilus*
T5	2% SPAE	*L. acidophilus*

**Table 3:** Change in pH of synbiotic yogurt sauce during storage.

Treatment	Day					
	0	7	14	21	28	35
T1	4.54 ± 0.04^a^	4.43 ± 0.02^a^	4.36 ± 0.03^a^	4.32 ± 0.02^a^	4.24 ± 0.03^a^	4.19 ± 0.01^a^
T2	4.52 ± 0.03^a^	4.40 ± 0.01^a^	4.30 ± 0.01^b^	4.18 ± 0.02^b^	4.08 ± 0.02^b^	4.01 ± 0.03^b^
T3	4.55 ± 0.01^a^	4.40 ± 0.02^a^	4.25 ± 0.03^c^	4.15 ± 0.02^b^	4.03 ± 0.01^c^	3.95 ± 0.02^c^
T4	4.56 ± 0.03^a^	4.37 ± 0.01^a^	4.24 ± 0.01^c^	4.12 ± 0.00^c^	4.00 ± 0.02^c^	3.92 ± 0.03^c^
T5	4.54 ± 0.02^a^	4.30 ± 0.03^b^	4.16 ± 0.05^d^	4.01 ± 0.02^d^	3.91 ± 0.01^d^	3.80 ± 0.01^d^

Different lowercase letters indicate significant differences between groups (*P* < 0.05).

**Table 4:** Change in acidity (g/L) of synbiotic yogurt sauce during storage.

Treatment	Day					
	0	7	14	21	28	35
T1	0.74 ± 0.01^a^	0.76 ± 0.02^a^	0.77 ± 0.01^a^	0.77 ± 0.00^a^	0.78 ± 0.02^a^	0.79 ± 0.01^a^
T2	0.75 ± 0.02^a^	0.78 ± 0.02^a^	0.82 ± 0.01^a^	0.85 ± 0.01^b^	0.86 ± 0.01^b^	0.87 ± 0.00^b^
T3	0.73 ± 0.02^a^	0.78 ± 0.01^a^	0.84 ± 0.00^a^	0.86 ± 0.02^b^	0.88 ± 0.01^b^	0.88 ± 0.00^b^
T4	0.74 ± 0.01^a^	0.80 ± 0.02^a^	0.85 ± 0.01^a^	0.86 ± 0.02^b^	0.89 ± 0.01^b^	0.90 ± 0.02^b^
T5	0.72 ± 0.00^a^	0.81 ± 0.02^a^	0.88 ± 0.01^a^	0.94 ± 0.02^c^	0.92 ± 0.02^b^	0.98 ± 0.01^c^

Different lowercase letters indicate significant differences between groups (*P* < 0.05).

**Table 5:** Change in viscosity (Pa/s) of synbiotic yogurt sauce during storage.

Day	Treatment				
	T1	T2	T3	T4	T5
0	265.21 ± 2.01^c^	261.42 ± 3.03^d^	257.13 ± 1.54^e^	269.14 ± 4.02^b^	279.44 ± 2.42^a^
35	219.25 ± 1.90^a^	189.27 ± 2.04^b^	162.71 ± 4.02^c^	148.15 ± 3.09^d^	112.31 ± 1.85^e^

Different lowercase letters indicate significant differences between groups (*P* < 0.05).

**Table 6:** Change in color values of synbiotic yogurt samples.

Parameter	Treatment				
	T1	T2	T3	T4	T5
*L*	51.01 ± 0.04^a^	50.92 ± 0.06^a^	51.03 ± 0.11^a^	51.09 ± 0.07^a^	50.84 ± 0.11^a^
*a*	3.18 ± 0.05^a^	3.32 ± 0.04^b^	12.71 ± 0.13^c^	16.15 ± 0.19^d^	21.31 ± 0.05^e^
*b*	9.77 ± 0.08^a^	9.82 ± 0.03^a^	10.01 ± 0.06^b^	10.12 ± 0.05^b^	9.79 ± 0.09^a^
Δ*E*	1.05 ± 0.08^a^	1.07 ± 0.04^a^	1.91 ± 0.12^b^	2.56 ± 0.09^c^	4.71 ± 0.15^d^

Different lowercase letters indicate significant differences between groups (*P* < 0.05).

## Data Availability

The data used to support the findings of this study are included within the article.
